# Diagnostic value of MIBG cardiac scintigraphy for differential dementia diagnosis

**DOI:** 10.1002/gps.4229

**Published:** 2014-11-03

**Authors:** Sylvie Slaets, Frank Van Acker, Jan Versijpt, Lothar Hauth, Johan Goeman, Jean-Jacques Martin, Peter Paul De Deyn, Sebastiaan Engelborghs

**Affiliations:** 1 Reference Center for Biological Markers of Dementia (BIODEM), and Biobank, Institute Born-Bunge, University of AntwerpAntwerp, Belgium; 2 Department of Nuclear Medicine, Hospital Network Antwerp (ZNA) MiddelheimAntwerp, Belgium; 3 Department of Neurology, University Hospital BrusselsBrussels, Belgium; 4 Department of Neurology and Memory Clinic, Hospital Network Antwerp (ZNA) Middelheim and Hoge BeukenAntwerp, Belgium; 5 Department of Neurology and Alzheimer Research Center, University of Groningen and University Medical Center Groningen (UMCG)Groningen, The Netherlands

**Keywords:** dementia, dementia with Lewy bodies, Alzheimer’s disease, MIBG cardiac scintigraphy, sensitivity, specificity

## Abstract

**Objective:**

Iodine-123 metaiodobenzylguanidine (MIBG) cardiac scintigraphy has shown the potential to discriminate dementia with Lewy bodies (DLB) from Alzheimer’s disease (AD). However, these studies did not reflect clinical practice, as patients with ischemic heart disease, heart failure, diabetes mellitus, arterial hypertension, and hyperlipidemia and patients treated with antidepressants like trazodone were excluded.

**Methods:**

This study aimed to evaluate the use of MIBG cardiac scintigraphy to diagnose DLB in clinical practice. Moreover, the potential diagnostic value of MIBG cardiac scintigraphy in patients with clinically ambiguous dementia diagnosis (DLB versus AD) was tested. Eighty-five patients with a possible clinical diagnosis of DLB entered the study. MIBG uptake was determined by calculating the heart-to-mediastinum-uptake ratio (H/M).

**Results:**

The average H/M ratio was 1.42 ± 0.35. The number of core features for DLB and the H/M ratio were negatively correlated (*p* = 0.001; *r* = −0.360). With an H/M ratio cutoff of 1.68 in 20 patients with clinically ambiguous dementia diagnoses (DLB versus AD) at the moment of MIBG cardiac scintigraphy, 95% (19/20) of the patients were correctly classified as compared with clinical or definite diagnosis at follow-up, with sensitivity and specificity values for diagnosing DLB of 100% (16/16) and 75% (3/4), respectively. The H/M ratio was influenced only by age (*p* = 0.046; *r* = −0.217) and gender (*p* = 0.024) and not by any other variable studied.

**Conclusions:**

The MIBG cardiac scintigraphy H/M ratio is a possible diagnostic biomarker for DLB in routine clinical practice and might have an added diagnostic value in case of doubt between DLB and AD. Copyright © 2014 John Wiley & Sons, Ltd.

## Introduction

Dementia with Lewy bodies (DLB) is the second most common cause of neurodegenerative dementia in older people after Alzheimer’s disease (AD). Given the overlap in clinical symptoms between DLB and AD, differential diagnosis is difficult (Merdes *et al*., 2003). Although the cerebrospinal fluid (CSF) biomarkers Aβ_1–42_, T-tau and P-tau_181P_ have an added diagnostic value for differential dementia diagnosis, concomitant amyloid pathology in DLB limits the use of CSF Aβ_1–42_ for discriminating DLB from AD (Slaets *et al*., 2013). However, discrimination between DLB and AD is clinically relevant, as there is a difference in pharmacological treatment options (e.g., severe adverse effects to antipsychotic drugs in DLB patients). Moreover, an early and accurate (differential) dementia diagnosis will become more and more important once disease-modifying drugs for AD or other neurodegenerative brain diseases will become available, as these (potentially toxic) drugs will probably be pathology specific.

Iodine-123 metaiodobenzylguanidine (MIBG) cardiac scintigraphy has been proposed as a method to differentiate between DLB and AD. The uptake and storage in presynaptic vesicles of MIBG is probably identical to norepinephrine (Wieland *et al*., 1980). MIBG is released into the synaptic cleft but is not metabolized. The decreased cardiac uptake of MIBG in DLB reflects the postganglionic cardiac sympathetic denervation (Orimo *et al*., 2005). Promising results have been published, but these studies have been conducted under very strict exclusion criteria (Yoshita *et al*., 2001, Yoshita *et al*., 2006, Watanabe *et al*., 2001, Oide *et al*., 2003, Hanyu *et al*., 2006, Wada-Isoe *et al*., 2007). Indeed, patients with ischemic heart disease, heart failure, thyroid disease, or diabetes mellitus were excluded (Yoshita *et al*., 2001, Yoshita *et al*., 2006, Watanabe *et al*., 2001, Oide *et al*., 2003). Moreover, patients with arterial hypertension or hyperlipidemia (Yoshita *et al*., 2001, Yoshita *et al*., 2006) and patients on antidepressant medication like trazodone or on antipsychotics like haloperidol were excluded from these studies (Yoshita *et al*., 2001, Yoshita *et al*., 2006, Watanabe *et al*., 2001, Oide *et al*., 2003). As most patients with a cognitive deterioration are older people having comorbidities like arterial hypertension, diabetes mellitus, cardiovascular disease, thyroid disease, or hyperlipidemia, these studies do not reflect clinical practice.

This study aimed to evaluate the use of MIBG cardiac scintigraphy to diagnose DLB in clinical practice. Moreover, the potential diagnostic value of MIBG cardiac scintigraphy in patients with clinically ambiguous dementia (DLB versus AD) diagnosis was tested.

## Methods

The study population consisted of patients visiting the memory clinic between 2006 and 2013 and in whom DLB was considered as a possible clinical diagnosis (*n* = 85). For all patients, the presence of core features and suggestive features for DLB, as determined by McKeith *et al*. (McKeith *et al*., 2005), were recorded. Relevant concomitant diseases and conditions like diabetes mellitus, arterial hypertension, hyperlipidemia, ischemic heart disease, and heart failure as well as pharmacological treatments at the time of MIBG scanning were recorded.

Patients were injected with MIBG (2 mCi), and images were acquired 4 h after injection. The data of 67 patients were acquired with a Philips XCT scanner, whereas for 18 patients, the data were acquired with a Varicam (GE) scanner, both with a low-energy, high-resolution collimator. Both cameras had similar hardware characteristics (LEHR collimator, large field double-head camera) and the same settings of acquisition parameters (energy spectrum, pixel size, scan duration). Thyroid blockage was acquired with potassium perchlorate. MIBG uptake was determined by calculating the heart-to-mediastinum-uptake (H/M) ratio. By applying the H/M ratio, potential differences due to the use of two different cameras were minimized. All the scans were analyzed by the same nuclear medicine physician (FVA) who drew the regions of interest and evaluated the scans.

Patients were followed up at the memory clinic. The physicians who clinically followed up the patients were not blinded to the MIBG results at baseline. At follow-up, the clinical diagnosis of probable AD was made according to the National Institute of Neurological and Communicative Disorders and Stroke - Alzheimer’s Disease and Related Disorders Association (NINCDS-ADRDA) criteria (McKhann *et al*., 1984). Probable DLB was diagnosed according to the criteria of McKeith *et al*. (McKeith *et al*., 2005). In case consenting patients died, autopsy was performed in order to establish a definite dementia diagnosis. For the neuropathological diagnosis of AD, the criteria of Braak and Braak (1991) and Braak *et al*. (2006) were applied as described earlier (Le Bastard *et al*., 2013).

Diagnostic accuracy of MIBG cardiac scintigraphy was tested as compared with the definite or clinical dementia diagnosis at follow-up. A *t*-test was used to evaluate the difference with regard to the H/M ratio between patients with and without diabetes mellitus, possibly interfering medication, arterial hypertension, and hyperlipidemia. To examine the relationship between the H/M ratio and age, the number of core and suggestive features of DLB, a Pearson’s correlation coefficient was executed. A significance level of <0.05 was considered significant. Statistical analyses were performed using SPSS 20 (SPSS Inc., Chicago, USA).

This study was approved by the ethics committee of the University of Antwerp, Antwerp, Belgium.

## Results

Data of 85 patients with a possible clinical diagnosis of DLB were available for data analyses. The average age of the population was 76 ± 8 years. The population consisted of 52 males and 33 females. The average MMSE score was 20 ± 6 (*n* = 84). Ten patients had diabetes mellitus type II, and 10 patients suffered from a thyroid dysfunction. These conditions were treated in all patients. Twenty-two patients were treated with trazodone for diurnal rhythm disturbances, and two patients were treated with haloperidol. Twenty-seven patients presented with arterial hypertension, and 17 patients presented with hyperlipidemia. Nine patients had ischemic heart disease, and three patients had both heart failure and ischemic heart disease.

**Figure 1:**
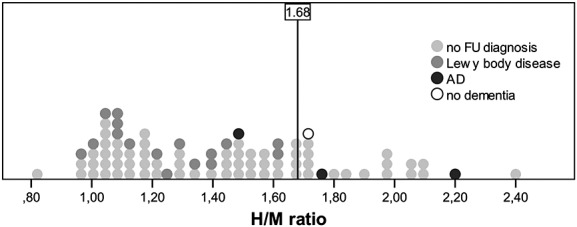
H/M ratios for the total population. The average H/M ratio was 1.42 ± 0.35. The H/M ratio cutoff of 1.68 of Yoshita *et al*. (Yoshita *et al*., 2006) to distinguish between AD and DLB is shown.

The H/M ratios for the population are represented in Figure1. The average H/M ratio was 1.42 ± 0.35. The H/M ratio was lower in males (1.35 ± 0.37) when compared with that in females (1.51 ± 0.29) (*p* = 0.024). The H/M ratio was not significantly lower in patients treated with trazodone (*p* = 0.908). No significant difference could be found in patients with diabetes mellitus type II, arterial hypertension, heart failure/ischemic heart disease, hyperlipidemia, and thyroid dysfunction (*p* = 0.171; *p* = 0.104; *p* = 0.104; *p* = 0.349; *p* = 0.106). A significant negative correlation was found between age and the H/M ratio (*p* = 0.046; *r* = −0.217).

**Figure 2:**
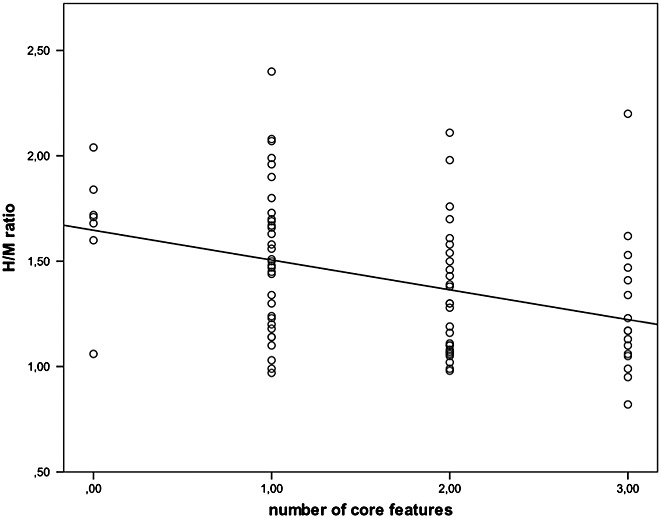
Correlation between number of core features of DLB and the H/M ratio (*p* = 0.001; *r* = −3.60).

A negative correlation between the number of core features for DLB and the H/M ratio (*p* = 0.001; *r* = −0.360) was found (Figure2). No significant correlation was found between disease duration and the H/M ratio (*p* = 0.286) or between the number of suggestive features and the H/M ratio (*p* = 0.060).

The MIBG classification and the clinical or definite dementia diagnosis at follow-up were compared for patients with clinically ambiguous diagnoses (AD or DLB) at baseline and either one of the following: (i) clinical follow-up of more than six months after MIBG cardiac scintigraphy (*n* = 19) or (ii) autopsy confirmation of the clinical diagnosis (*n* = 1) (Table1). The clinical diagnosis at last follow-up or the definite dementia diagnosis was used to test the diagnostic accuracy of the MIBG H/M ratio at baseline. The mean follow-up term was 17 ± 14 months (range: 6–57 months) (Table1). One patient had an autopsy-confirmed diagnosis of AD (case no. 5, Table1). His H/M ratio was 1.76. This patient had Braak stage III–IV for neurofibrillary tangles, and no Lewy bodies were found. Based on the literature (Yoshita *et al*., 2006), an H/M ratio cutoff of 1.68 was used for the classification of patients based on MIBG cardiac scintigraphy. Applying the H/M ratio cutoff of 1.68, 95% (19/20) of the patients with clinically ambiguous diagnoses were correctly classified, with sensitivity and specificity values of 100% (16/16) and 75% (3/4), respectively, as compared with diagnosis at follow-up.

**Table 1:** Patients with clinically ambiguous dementia diagnoses (DLB versus AD) at the moment of MIBG cardiac scintigraphy

Subject number	Gender	Age	Follow-up (months)	H/M ratio	Possibly interfering medication	Cardiovascular comorbidity, LVEF (if available)	Follow-up diagnosis	Core features of DLB	Suggestive features of DLB
**1**	M	73	7	1.71	–	Hyperlipidemia, AHT, IHD, LVEF 59%	No dementia		
**2**	F	81	7	1.41	–	AHT, LVEF 60%	DLB	F/H/P	
**3**	M	79	10	1.10	–		DLB	F/H/P	
**4**	M	73	6	1.30	–	AHT, IHD, heart failure	DLB	H/P	Positive DAT scan
**5**	M	78	1	1.76	–	–	Definite AD	H/P	
**6**	M	87	11	1.62	–	–	DLB	F/H/P	
**7**	M	77	8	1.47	Trazodone	LVEF 63%	AD with CVD	H	
**8**	M	78	19	1.05	–		DLB	F	
**9**	M	76	21	1.10	–	IHD, heart failure	DLB	H/P	
**10**	F	83	17	1.24	–	–	DLB	P	Severe neuroleptic sensitivity
**11**	F	75	17	1.38	–	AHT, AF, myocardial hypertrophy, LVEF 65%	DLB	F	
**12**	M	71	15	1.34	Trazodone	AHT, LVEF 70%	DLB	F/H/P	
**13**	M	72	8	1.23	–	AHT, AF	DLB	P	
**14**	M	72	44	2.20	–	–	AD	F/H/P	Severe neuroleptic sensitivity
**15**	F	81	6	0.97	–	–	DLB	P	Positive DAT scan
**16**	F	79	18	1.44	–	–	AD + PD	P	
**17**	M	84	31	1.11	Trazodone	Hyperlipidemia, AHT	DLB	F	Positive DAT scan and REM sleep behavior disorder
**18**	M	75	6	1.61	Trazodone	–	DLB	H/P	Severe neuroleptic sensitivity
**19**	M	74	13	0.99	Trazodone	Hyperlipidemia, IHD, AF	DLB	F/H/P	
**20**	M	75	57	1.07		–	PDD	F/H/P	REM sleep behavior disorder

Abbreviations: AF = atrial fibrillation; AHT = arterial hypertension; DLB = dementia with Lewy bodies; H = visual hallucinations; IHD = ischemic heart disease; F = fluctuating cognition; LVEF = left ventricular ejection fraction; P = Parkinsonism; DAT = dopamine transporter imaging; CVD = cerebrovascular disease; PD = Parkinson’s disease.

## Discussion

Previous research has shown promising results for the use of MIBG cardiac scintigraphy as a diagnostic tool to distinguish DLB from AD (Yoshita *et al*., 2001, Yoshita *et al*., 2006, Watanabe *et al*., 2001, Oide *et al*., 2003, Hanyu *et al*., 2006, Wada-Isoe *et al*., 2007) with sensitivity and specificity values of 100% (Yoshita *et al*., 2006). However, most studies included limited study populations, and studies in Caucasian populations are sparse. Moreover, in former studies, the populations consisted of patients with a probable clinical diagnosis of AD or of DLB. Also, patients with diabetes mellitus, arterial hypertension, heart failure, cardiac ischemic heart disease, or hyperlipidemia were excluded from these studies as well as patients under pharmacological treatment (like trazodone or haloperidol) that could influence MIBG uptake (Solanki *et al*., 1992). In our study population, 62% of the patients (53/85) would have been excluded if the exclusion criteria of previously published papers were applied. Nevertheless, in clinical practice, there often is diagnostic doubt between DLB and AD, given the overlap in clinical symptoms as well as an AD CSF biomarker profile that is often encountered in DLB patients (Slaets *et al*., 2013). In the current study, we wanted to evaluate the use of MIBG cardiac scintigraphy for diagnosing DLB in clinical practice, not excluding patients with diabetes mellitus, ischemic heart disease, heart failure, arterial hypertension, or hyperlipidemia or patients treated with medication that could possibly influence MIBG uptake.

In the current study, a significant and negative correlation was found between the number of core features of DLB and the H/M ratio (*p* = 0.001; *r* = −0.360), suggesting that decreased H/M ratios are intrinsically related to the clinical features of DLB. Previous research from Kobayashi *et al*. (Kobayashi *et al*., 2009) has also found a relation between the H/M ratio and the clinical symptoms in DLB. In the latter study, a lower H/M ratio was found in DLB patients with orthostatic hypotension when compared with DLB patients without orthostatic hypotension. Although we detected a correlation with the number of core features, no correlation was found between the H/M ratio and disease duration, suggesting that low H/M ratios can be found early in the disease course, which is in accordance with several previous studies (Fujishiro *et al*., 2012, Sakakibara *et al*., 2012, Estorch *et al*., 2008).

Moreover, the potential diagnostic value of MIBG cardiac scintigraphy in patients with clinically ambiguous dementia (DLB versus AD) diagnoses was tested. Clinically ambiguous diagnoses resulted from diagnostic doubt between DLB and AD following routine clinical diagnostic workup. Applying the H/M ratio cutoff of 1.68 (Yoshita *et al*., 2006), the sensitivity and specificity for a diagnosis of DLB in this clinical population were 100% and 75%, respectively (Figure1). In the study of Yoshita *et al*. (Yoshita *et al*., 2006) that examined the use of the H/M ratio to distinguish between AD and DLB in however highly selected populations, a sensitivity and specificity value of 100% was achieved (Yoshita *et al*., 2006).

In this study, a negative correlation was found between age and H/M ratio (*p* = 0.046; *r* = −0.217). These results are consistent with other studies and are suggestive of a reduced neuronal reuptake of norepinephrine with aging (Sakata *et al*., 2009). The H/M ratio was also lower in men (*p* = 0.024). Differences in H/M ratios as a result of gender and age could possibly lead to false-positive results. Therefore, an age- and gender-corrected H/M ratio should be considered to increase the diagnostic accuracy of MIBG cardiac scintigraphy.

In previous studies, patients with comorbidities that might affect MIBG cardiac scintigraphy were excluded. In our population that reflects clinical practice, cardiovascular comorbidities and thyroid dysfunction had no significant effect on H/M ratios. In the present study, 22/85 patients were treated with trazodone at the moment of MIBG cardiac scintigraphy. Although a decrease in H/M ratio was theoretically to be expected due to the depletion of storage vesicles by trazodone (Solanki *et al*., 1992), we could not detect a difference in H/M ratios comparing patients treated with and without trazodone (*p* = 0.908). This finding is supported by previous in vitro research on rabbit myocardium where no influence of trazodone could be detected (Mayer *et al*., 2000).

Finally, a number of limitations need to be considered. The sensitivity and specificity values calculated in this study were based on a relatively small number of patients, because only patients with possible clinical diagnoses of DLB were included in this study, moreover resulting in few patients with other (than DLB) neurodegenerative brain disorders. The physicians who clinically followed up the patients were not blinded to the MIBG results at baseline. The H/M cutoff value that was used was based on literature although it has been shown that the optimal cutoff can vary between centers.

Further research through a large prospective, longitudinal study is needed to confirm the diagnostic potential of MIBG cardiac scintigraphy for DLB and to discriminate DLB from AD. Studies that contain patients with autopsy-confirmed dementia diagnoses (both DLB and other neurodegenerative dementias like AD) are especially needed to confirm the diagnostic potential of MIBG cardiac scintigraphy for differential dementia diagnosis and to gain more insight into factors of potential influence on the H/M ratio, like psychotropic medication and cardiovascular comorbidities.

## Conclusion

The MIBG cardiac scintigraphy H/M ratio is a possible diagnostic biomarker for DLB in routine clinical practice and might have an added diagnostic value in case of doubt between DLB and AD.

## Key points

- Given the overlap in clinical symptoms between DLB and AD, differential diagnosis is difficult.
- MIBG cardiac scintigraphy H/M ratio is a possible diagnostic biomarker for DLB.
- MIBG cardiac scintigraphy might have an added diagnostic value in case of doubt between DLB and AD.
